# Diabetes Impairs Angiogenesis and Induces Endothelial Cell Senescence by Up-Regulating Thrombospondin-CD47-Dependent Signaling

**DOI:** 10.3390/ijms20030673

**Published:** 2019-02-04

**Authors:** Milad S. Bitar

**Affiliations:** Department of Pharmacology& Toxicology, Faculty of Medicine, Kuwait University, P.O. Box 24923, Safat 13110, Kuwait; milad.bitar@gmail.co; Tel.: +965-6641-7504; Fax: +965-2531-8454

**Keywords:** endothelial dysfunction, impaired angiogenesis, diabetes, cellular senescence TSP1-CD47, oxidative stress.

## Abstract

Endothelial dysfunction, impaired angiogenesis and cellular senescence in type 2 diabetes constitute dominant risk factors for chronic non-healing wounds and other cardiovascular disorders. Studying these phenomena in the context of diabetes and the TSP1-CD-47 signaling dictated the use of the in vitro wound endothelial cultured system and an in vivo PVA sponge model of angiogenesis. Herein we report that diabetes impaired the in vivo sponge angiogenic capacity by decreasing cell proliferation, fibrovascular invasion and capillary density. In contrast, a heightened state of oxidative stress and elevated expression of TSP1 and CD47 both at the mRNA and protein levels were evident in this diabetic sponge model of wound healing. An in vitro culturing system involving wound endothelial cells confirmed the increase in ROS generation and the up-regulation of TSP1-CD47 signaling as a function of diabetes. We also provided evidence that diabetic wound endothelial cells (W-ECs) exhibited a characteristic feature that is consistent with cellular senescence. Indeed, enhanced SA-β-gal activity, cell cycle arrest, increased cell cycle inhibitors (CKIs) p53, p21 and p16 and decreased cell cycle promoters including Cyclin D1 and CDK4/6 were all demonstrated in these cells. The functional consequence of this cascade of events was illustrated by a marked reduction in diabetic endothelial cell proliferation, migration and tube formation. A genetic-based strategy in diabetic W-ECs using CD47 siRNA significantly ameliorated in these cells the excessiveness in oxidative stress, attenuation in angiogenic potential and more importantly the inhibition in cell cycle progression and its companion cellular senescence. To this end, the current data provide evidence linking the overexpression of TSP1-CD47 signaling in diabetes to a number of parameters associated with endothelial dysfunction including impaired angiogenesis, cellular senescence and a heightened state of oxidative stress. Moreover, it may also point to TSP1-CD47 as a potential therapeutic target in the treatment of the aforementioned pathologies.

## 1. Introduction

Type 2 diabetes (T2D) has emerged as a major threat to human health span, especially in the face of a rapidly growing aging population and increasing prevalence of obesity. Diabetes incidence and prevalence increase as a function of age, with about 25.9% of Americans 65 years or older having diabetes as compared to 9.3% in the general population [1]. This disease state constitutes a major risk factor for premature onset of a number of age-related conditions including cardiovascular disease, stroke, infection and impaired wound healing [2,3,4]. Diabetics also appear to have both higher mortality and an increased rate of amputation following acute limb ischemia or foot ulcers [5]. Altered angiogenesis and endothelial dysfunction are common features of T2D and they may contribute to some of the aforementioned abnormalities [6,7,8]. Indeed, an aberrant endothelial activation with reduced proliferation and migration has been demonstrated in wound angiogenesis during the course of diabetes, and ulcers of ischemia and venous insufficiency [3,9]. Similarly, reduced mobilization of bone marrow-derived endothelial progenitor cells to the wound milieu was also evident in these disease states [10,11]. To this end, delineating the pathogenetic mechanism(s) responsible for diabetes-related impairment of angiogenesis and endothelial function should have important implications for understanding and treating the various forms of cardiovascular disease and wound healing defect.

Cellular senescence, an essentially irreversible growth arrest of a cell, is emerging as a fundamental mechanism contributing to tissue dysfunction and many age- or diabetes-related pathologies including CVD [1]. This phenomenon can occur in response to a plethora of stressors such as DNA damage, telomere dysfunction [12,13], ionizing radiation [14], high glucose concentrations [15], low-grade inflammation [16] or oxidative stress [17]. Senescent cells are usually featured by the up-regulation of cell cycle inhibitors such as p53, p21 and p16, accumulation of DNA damage foci, heightened state of oxidative stress and enhanced β-galactosidase (SA-β-gal) activity [18]. Although these cells are incapable of dividing, they are metabolically active and support the release of pro-inflammatory and pro-oxidant molecules collectively known as senescent associated secretory phenotype (SASP) [19].

Senescent ECs, an early marker of vascular abnormalities, exhibit reduced generation of the key vasodilatory and vasoprotective molecule nitric oxide (NO) together with enhanced production of reactive oxygen species (ROS) and decrease in the cell repairing mechanism [20,21,22,23]. These cells are implicated in a number of cardiovascular dysfunctions that are commonly seen in experimental and clinical diabetes including, arterial stiffing/remodeling [24], impaired angiogenesis/wound healing [25] and increased in the incidence of atherosclerosis. In view of the above information, it is reasonable to suggest that the diabetic microenvironment could be permissive to the development and accumulation of senescent cells. On the other hand, senescent cells may contribute to tissue dysfunction and comorbidities observed in T2D. Overall, identifying the underlying mechanisms of endothelial cell senescence may help to draw a common pathway unifying endothelial dysfunction to cardiovascular disorders and diabetic complications (e.g., impaired angiogenesis). Moreover, the phenomenon of senescence can be illuminated as a target for novel therapy that culminates in improving quality of life through rejuvenation of several organs including the vascular system.

Thrombospondin-1 (TSP1) is often secreted at sites of injury and tissue remodeling by many cell types including fibroblasts and endothelial cells [26]. This matricellular protein is a well-known inhibitor of angiogenesis and this antiangiogenic effect is mediated by the binding of TSP1 to cell surface receptors, namely CD36 and CD47 with a consequent suppression of key angiogenic markers including the nitric oxide (NO)/c-GMP-dependent signaling [27,28]. CD47 is a ubiquitous transmembrane receptor that serves not only as a sensor for cell-cell and cell-microenvironment signal, but also as a ligand for signal regulatory protein-α and TSP1. The TSP1-CD47 axis participates in important cellular processes including angiogenesis and atherosclerotic response [26,29]. More recent data have advanced the notion that lack of CD47 expression in endothelial cells enabled them to spontaneously gain characteristics consistent with embryonic stem cells [30]. Furthermore, TSP1 significantly accelerated replicative senescence and associated cell cycle arrest in a CD47-dependent manner [31]. We recognize that most of these previous studies used endothelial cells that were not exposed to an elaborate set of micro-environmental cues ex vivo. For example, the fluid bathing the wound tissue reflects the wound microenvironment and shapes the functional response of wound-related cells, such as endothelial cells [32]. This gap was addressed by isolating intact endothelial cells from the actual wound milieu of subcutaneous sponge implants, a well-established in vivo model of angiogenesis. The current study was initiated to highlight the role and mechanisms of TSP1-CD47-dependent signaling in regulating angiogenesis, cell cycle progression, and cellular senescence in wound endothelial cells of T2D.

## 2. Results

### 2.1. GK Diabetic W-ECs Showed Low Proliferation Rate, Decreased Survival and Increased Apoptosis

**Figure 1:**
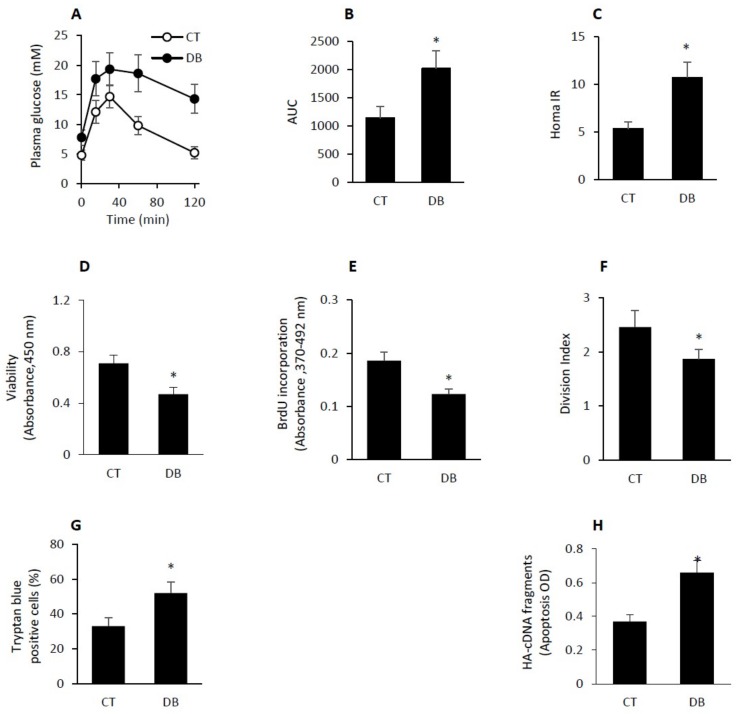

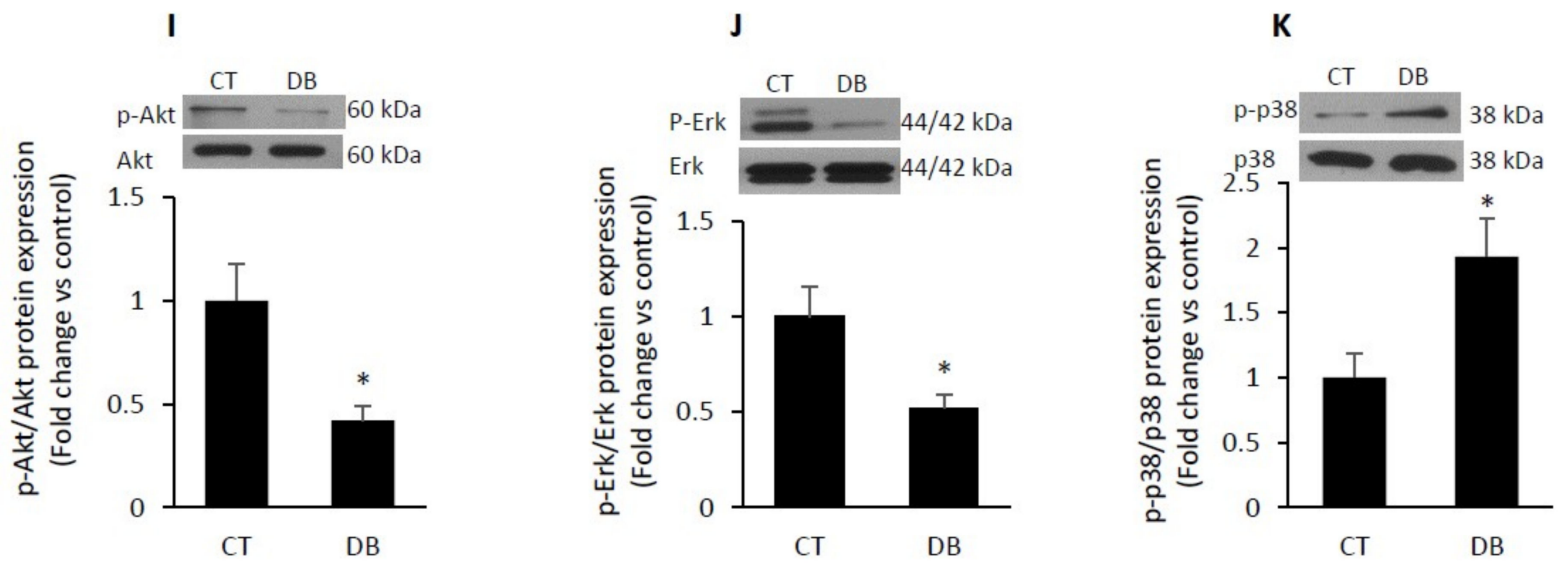
Diabetic W-ECs showed low proliferation rate, decreased survival and increased apoptosis. (**A**) GK rats had impaired glucose tolerance (e.g., higher AUC over 2 h) following i.p. glucose administration (2 g/kg) when compared with Wistar controls. (**B**) HOMA-IR-assessed by fasting plasma glucose and insulin levels was higher in GK rats, indicating insulin resistance. (**C**) Cell viability was determined in diabetic and control W-ECs using WST-1-based assay. (**D**) Rate of proliferation was quantified by measuring BrdU incorporation into W-ECs. (**E**) Division index of diabetic and control W-ECs was measured by CFSE dilution. (**F**) Trypan blue staining and HA-cDNA fragments (**G**) were used to detect the rate of apoptosis in both diabetic and control W-ECs. (**H**–**K**) Key signaling molecules involved in cell survival, p-Akt/Akt, cell proliferation, p-ERK/ERK and cell apoptosis, p-p38/p38 were quantified using a Western blotting-based technique. Abbreviations: W-ECs, wound endothelial cells; AUC, area under the curve; HOMA-IR, homeostasis model assessment of insulin resistance; WST, 4-[3-(4-Iodophenyl)-2-(4-nitrophenyl)-2H-5-tetrazolio]-1,3-benzene Disulfonate; HA-cDNA; cytoplasmic histone-associated DNA fragments; BrdU, bromodeoxyuridine; CT, control; DB, diabetic. Values are means ± SEM (*n* = 6) * Significantly different from corresponding control values at *p* ≤ 0.05.

Subcutaneously implanted polyvinyl alcohol (PVA) sponges into female Wistar control and Goto-Kakizaki (GK; age 12–15 months) rats were used to study the in vivo and in vitro cellular senescence and angiogenic activity. The GK rats showed moderate elevation in plasma glucose level (control, 4.92 ± 0.51 mM vs. diabetic 7.87 ± 0.85 mM), impaired glucose tolerance (Figure 1A,B) and decreased insulin sensitivity (Figure 1C); determined by the so called homeostasis model assessment of insulin resistance (HOMA-IR). In addition, others have reported that these animals also develop as a function of time chronic complications such as peripheral neuropathy [33], nephropathy [34] and retinopathy [35]. Most of the aforementioned pathogentic features are very similar to those seen in human disease and thus make the GK rats an ideal model for studying comorbidities of type 2 diabetes.

Primary wound endothelial cells (W-ECs) were isolated from control and diabetic PVA sponges, cultured in vitro for 6-day and then passaged and used (at passage 4–6) in various assays including cell viability, proliferation and apoptosis. As for the assessment of cell viability, we used 4-[3-(4-Iodophenyl)-2-(4-nitrophenyl)-2H-5-tetrazolio]-1,3-benzeneDisulfonate(WST1)- based technique and showed that diabetic W-ECs were less viable than corresponding control values (Figure 1D). Similarly, these cells also exhibited a significant decrease in bromdeoxyuridine (BrdU) uptake and carboxyfluorescein succinimidyl ester (CFSE) dilution indicating that cellular proliferation and the division index were also reduced as a function of diabetes (Figure 1E,F). In contrast, trypan blue positive cells and cytoplasmic histone-associated DNA (HA-DNA) fragments, markers for apoptotic cell death, were elevated in diabetic W-ECs as compared to control W-ECs (Figure 1G,H).

To explore the cellular mechanisms responsible for the decrease in cell proliferation/ survival and the increase in apoptotic cell death in diabetic W-ECs, we measured the levels of p-Akt, p-ERK and p-p38 since these signaling pathways have been shown to be involved in cell survival, proliferation and apoptosis, respectively. Our data revealed that the ratios of p-AKT/Akt and p-ERK/ERK were markedly decreased in diabetic W-ECs relative to control W-ECs (Figure 1I,J). In contrast, an increase in p-p38/p38 level was evident in these cells (Figure 1K). 

### 2.2. Diabetes Suppresses Angiogenic Capacity in W-ECs

**Figure 2:**
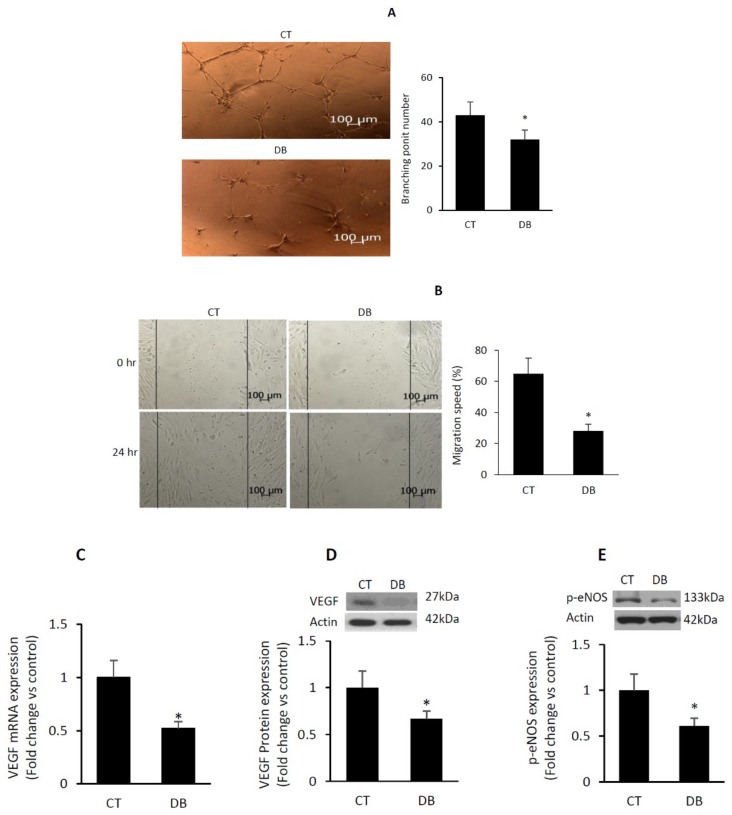
Diabetes suppresses angiogenic capacity in W-ECs. (**A**) Photomicrographs of tube formation of W-ECs that were seeded on growth factor-reduced Matrigel; accompanied by a barograph figure denoting the quantitative measure of the branching point number. (**B**) Photomicrographs of cell migration (e.g., scratch of cells with a pipette tip) followed by light microscope-based measurement of the distance of wound covered by cells; accompanied by a barograph figure indicating the quantitative measure of migration speed expressed as percent of closure. (**C**,**D**) VEGF expression in terms of mRNA and protein levels was measured using qRT-PCR and Western blotting-based techniques. (**E**) p-eNOS, a measure of eNOS activity, was determined by Western blotting. Abbreviation: W-EC, wound endothelial cells; VEGF, vascular endothelial growth factor; p-eNOS, phospho-endothelial nitric oxide synthase; CT, control; DB, diabetic. Values are means ± SEM (*n* = 6) * Significantly different from corresponding control values at *p* ≤ 0.05.

In vitro angiogenic potential of diabetic W-ECs versus control W-ECs was determined using a number of key events involved in angiogenesis, including proliferation, migration and tube formation. As indicated above, the rate of proliferation in diabetic W-ECs was suppressed, relative to control values (Please see Figure 1). Similarly, tube formation in term of branching point numbers and migration speed-in the wound healing assay were also decreased as a function of diabetes (Figure 2A,B).

Since VEGF is the most potent angiogenic growth factor, we assessed its levels in W-ECs both at the mRNA and protein levels using qRT-PCR and Western blotting, respectively. There was a 1.87-fold reduction in VEGF mRNA and a significant decrease in VEGF protein in diabetic vs. control W-ECs (Figure 2C,D). Similarly, a decrease in the level of p-eNOS (ser1177), an indicator of endothelial nitric oxide synthase activity (eNOS), was also evident in these cells (Figure 2E). 

### 2.3. Diabetes Impairs Angiogenic Activity in PVA Sponge Model of Wound Healing

**Figure 3:**
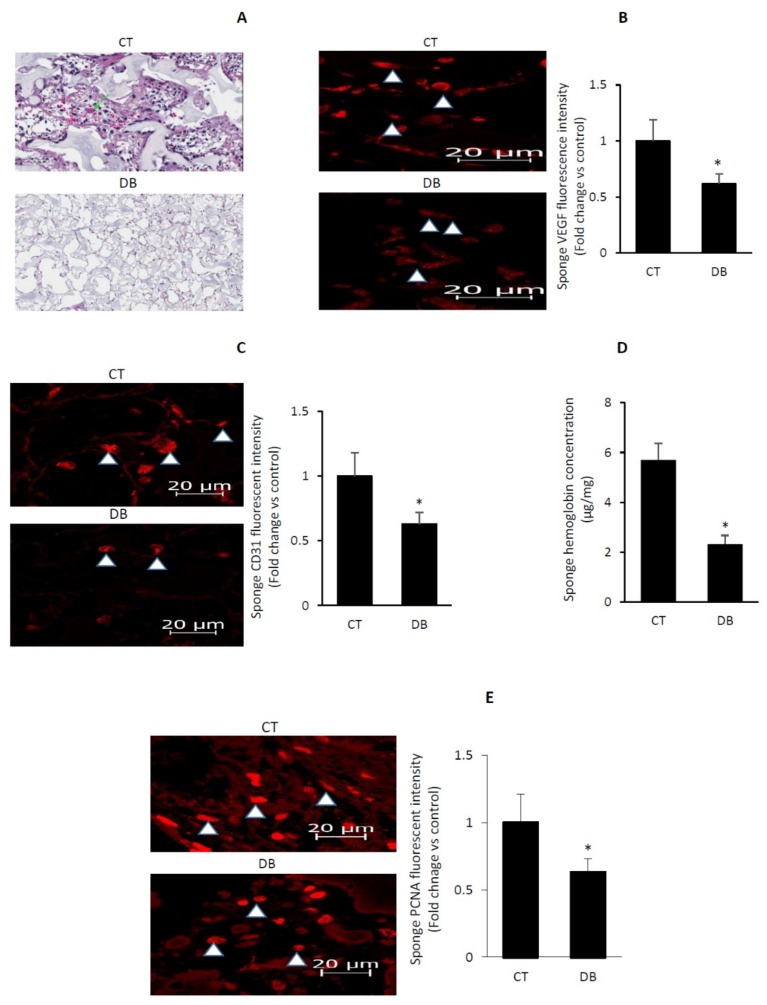
Diabetes suppresses angiogenic capacity in PVA sponge model of wound healing. (**A**) Representative photomicrographs of H&E stained sponge sections revealing fibrovascular invasion with the arrows denoting blood vessels containing red blood cells; magnification for CT is 60 µm and for DB is 200 µm. (**B**) Representative photomicrographs of VEGF immunofluorescence staining, accompanied by a barograph figure denoting the quantitative measure of VEGF fluorescence intensity. (**C**) Representative photomicrographs of CD 31 immunofluorescence staining, an indicator of microvascular density, accompanied by a barograph figure denoting the quantitative measure of CD 31 fluorescence intensity. (**D**) Hemoglobin contents in the sponges as determined by the Drabkin reagent. (**E**) Representative photomicrographs of PCNA immunofluorescence staining, an indicator of cell proliferation, accompanied by a barograph figure denoting the quantitative measure of PCNA fluorescence intensity. Abbreviation: PCNA, Proliferating Cell Nuclear Antigen, VEGF, vascular endothelial growth factor; CT, control; DB, diabetic. Values are means ± SEM (*n* = 6) * Significantly different from corresponding control values at *p* ≤ 0.05.

Next, we monitored the in vivo angiogenic capacity using a 10-day subcutaneously implanted PVA sponge model of wound healing. Hematoxylin/eosin-based staining revealed that sponges of T2D exhibited a decrease in the degree of fibrovascular invasion (Figure 3A). Moreover, using immunofluorescence microscopy, we confirmed a reduction in diabetic sponge contents of VEGF and CD31 (Figure 3B,C). Consistent with these data, a marked diminution in sponge level of hemoglobin was also evident in this disease state (Figure 3D). Since proliferation, like that of migration, constitutes an essential element of the angiogenic network, we assessed this process using the Proliferating Cell Nuclear Antigen (PCNA), a well-established marker of cell proliferation. Figure 3E showed that the fluorescence intensity of PCNA positive cells was less in sponges of T2D, as compared to corresponding control values. Together, the current findings denote that the processes involved in capillary formation in connection with blood vessel function are attenuated during the course of diabetes. Indeed, blood vessels indicated by arrows predominate in control but not in diabetic sponge sections (Figure 2A).

### 2.4. Diabetic W-ECs Exhibited a Characteristic Feature Consistent with Cellular Senescence and Cell-Cycle Arrest

**Figure 4:**
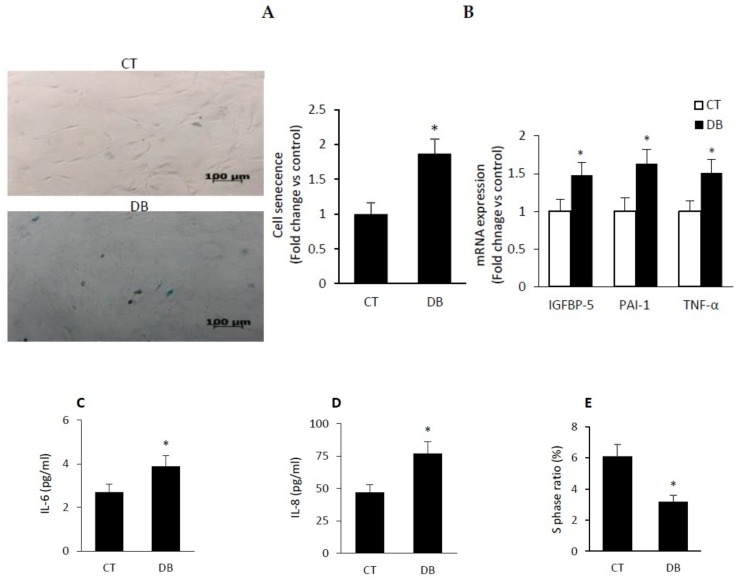

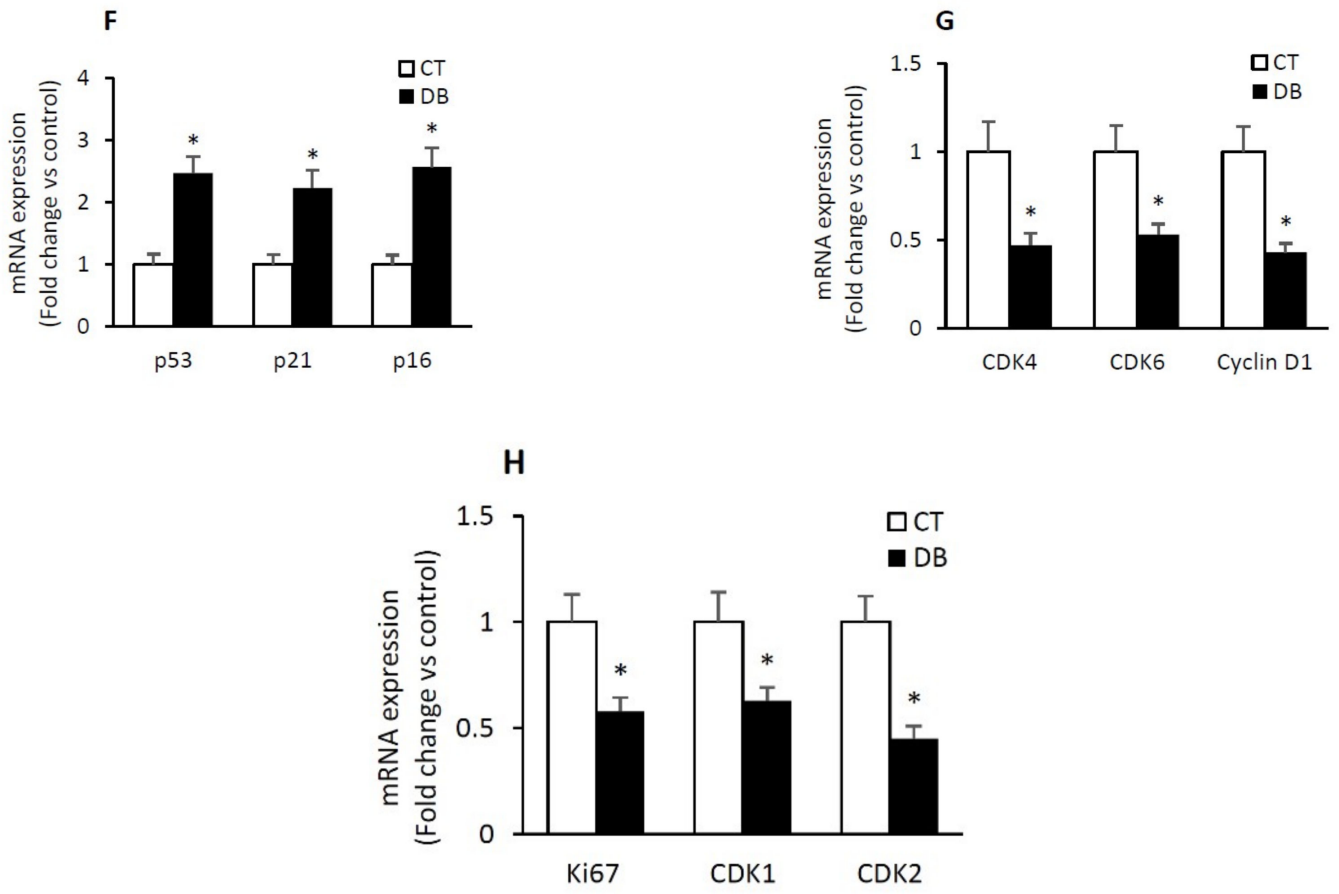
Diabetic W-ECs exhibited a characteristic feature consistent with cellular senescence and cell-cycle arrest. (**A**) Representative images of SA-β-gal staining (left; senescent cells stained with green); accompanied by a barograph figure denoting senescent cells as fold change vs. control. (**B**) mRNA levels of IGFBP5, PAI-1 and TNF-α quantified by qRT-PCR and expressed as a fold change vs. control. (**C**) IL-6 and IL-8 (**D**) in cultured medium of control and diabetic W-ECs quantified by ELISA and expressed as pg/mL. (**E**) Percentage of cells at the S phase measured using flow cytometry and cell cycle PI-based staining. Relative mRNA expression in control and diabetic W-ECs for cell cycle inhibitors (p53, p21, p16, (**F**)), cell cycle promoters (CDK4, CDK6, cyclin D1, (**G**)) and S-phase gene transcription (Ki67, CDK1, CDk2, (**H**)) expressed as a fold change vs. control. Abbreviation: TNF-α, tumor necrosis factor-alpha; IGFBP5, insulin-like growth factor binding protein5; PAI-1, prothrombotic-1; IL-6, interleukin-6; IL-8, interleukin-8, propodium iodide, CDK, cyclin dependent kinase; W-ECs, wound endothelial cells, CT, control; DB, diabetic. Values are means ± SEM (*n* = 6) * Significantly different from corresponding control values at *p* ≤ 0.05.

Assessment of diabetic W-ECs in the context of senescence dictated the measurement of senescence-associated β-galactosidase (SA-β-gal) activity, an indicator of cellular senescence, using fluorescence- or light microscope staining-based assay. The data revealed a clear increase in SA-β-gal positive cells in diabetic W-ECs at passage 6 as compared to control W-ECs (Figure 4A). Similarly, these cells also showed a feature of the senescence-associated secretory phenotype (SASP) as typified by the elevation in mRNA or protein levels of proinflammatory cytokines (TNF-α, IL-6 and IL-8), prothrombotic (PAI-1) and insulin-like growth factor binding protein 5 (IGF-BP5) (Figure 4B–D). These data harmonize well with previously published studies regarding premature senescence in endothelial cells and changes in gene expression of SASP [36,37].

Since cell cycle arrest, both in vivo and in vitro, is a well-established and indispensable marker of senescence [38], we assessed cell cycle distribution with the resulting data showing that the proportion of cells in the G1 phase was increased in diabetic W-ECs relative to those in control W-ECs (control W-ECs 74.5% vs. diabetic W-ECs, 87%). However, the percentage of cells in the S phase was markedly diminished as a function of diabetes (Figure 4E).

To further evaluate the molecular events involved in cellular senescence, we compared the mRNA levels of the key cell cycle inhibitors (CKIs) as in the case of p53, p21 and p16 that are known to be upregulated in senescent cells [39,40]. Consistent with the above cell cycle data (Figure 4E), the expression of mRNAs encoding for CKIs p53, p21, and p16 was significantly elevated in diabetic W-ECs relative to corresponding control W-ECs (Figure 4F). Contrastingly, mRNAs levels of cell cycle promoters including cyclin-dependent kinase (CDK) 4, (CDK4), CDK6 and Cyclin D1 showed marked reduction during the course of diabetes (Figure 4G). It is worthy of note that the above imbalance between CKIs and cell cycle promoters in diabetic W-ECs was associated with a significant decrease in E2F-mediated S-phase gene transcription such as Ki67, CDK1 and CDK2 (Figure 4H). Overall, these findings give credence to the concept that low-grade inflammation, cell-cycle arrest and cellular senescence are key phenotypic features of diabetic W-ECs.

### 2.5. Diabetes Induces a Heightened State of Oxidative Stress in Cultured W-ECs and In Vivo Sponge Model of Wound Healing

**Figure 5:**
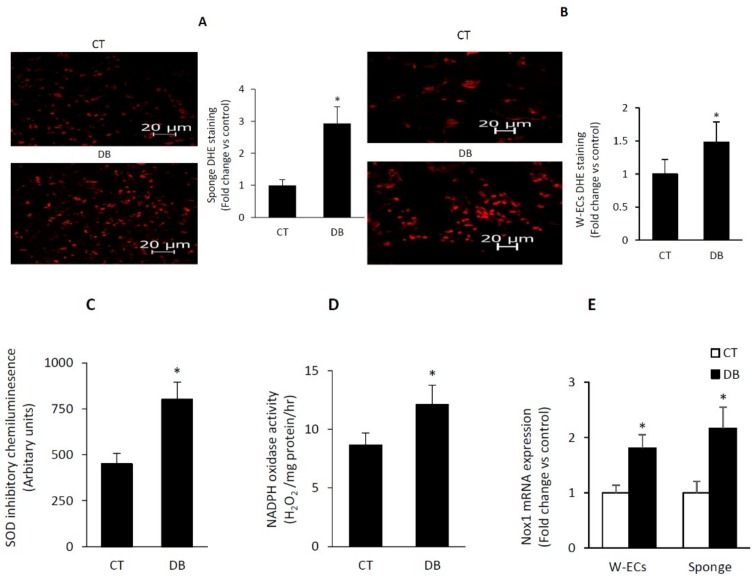
Diabetes induces a heightened state of oxidative stress in cultured W-ECs and in PVA sponge model of wound healing. Representative fluorescence images of DHE staining in W-ECs (**A**) and in PVA sponge model of angiogenesis (**B**); accompanied by a barograph figure denoting DHE staining as fold change vs. control. (**C**,**D**) NADPH oxidase activity in a membrane fraction of W-ECs was assessed using the lucigenin chemiluminescence- or the Amplex red/horseradish peroxidase fluorescence-based assay. (**E**) Nox1 mRNA expression in W-ECs and PVA sponge model of wound healing was determined using qRT-PCR-based assay. Abbreviation: DHE, dihydroethidium; SOD, superoxide dismutase; W-ECs, wound endothelial cells, CT, control; DB, diabetic. Values are means ± SEM (*n* = 6) * Significantly different from corresponding control values at *p* ≤ 0.05.

Having established that diabetic W-ECs exhibited features of cellular senescence and impaired angiogenesis, we sought to examine whether a common mechanism may be responsible for the development of these abnormalities. Our initial focus was on the ROS, since oxidative stress has been shown to contribute to endothelial dysfunction under various pathophysiological states [17]. Dihydroethidium (DHE) staining, an indicator of superoxide production, appears to be enhanced as a function of diabetes in vitro (W-ECs) and in vivo (PVA sponge) models of wound healing (Figure 5A,B). DHE-detected superoxide is a by-product of enzymatic oxidases and mitochondrial respiration and in this regard, we initially focused on NAD(P)H oxidase, a multi-protein enzyme complex, which is highly expressed in endothelial cells and uses NAD(P)H as a substrate to convert molecular oxygen to ROS [41]. Lucigenin chemiluminescence or the Amplex red/horseradish peroxidase fluorescence-based assays were used in the measurement of membranous NAD(P)H oxidase activity with the resulting data showing that diabetic W-ECs produced more superoxide and H_2_O_2_ than corresponding control W-ECs (Figure 5C,D). VAS 2870 was used as a specific NADPH oxidase inhibitor [42]. Consistent with these findings, we also confirmed in diabetic W-ECs a marked increase in the mRNA expression of Nox1 (Figure 5E), a major subunit of NADPH oxidase in the vascular system. Whether other forms including Nox2 and Nox4 are altered as a function of diabetes remain to be explored. To this end, our findings support the concept that in vitro cultured diabetic W-ECs, like that of in vivo sponge model of wound healing, retain their phenotypic feature of heightened state of oxidative stress even after 4–6 passages. This phenomenon is likely to stem, at least in part from the up-regulation of the NADPH oxidase system.

### 2.6. Diabetes Up-Regulates TSP1-CD47 System in Both Endothelial Cells and in PVA Sponge Model of Wound Healing

**Figure 6:**
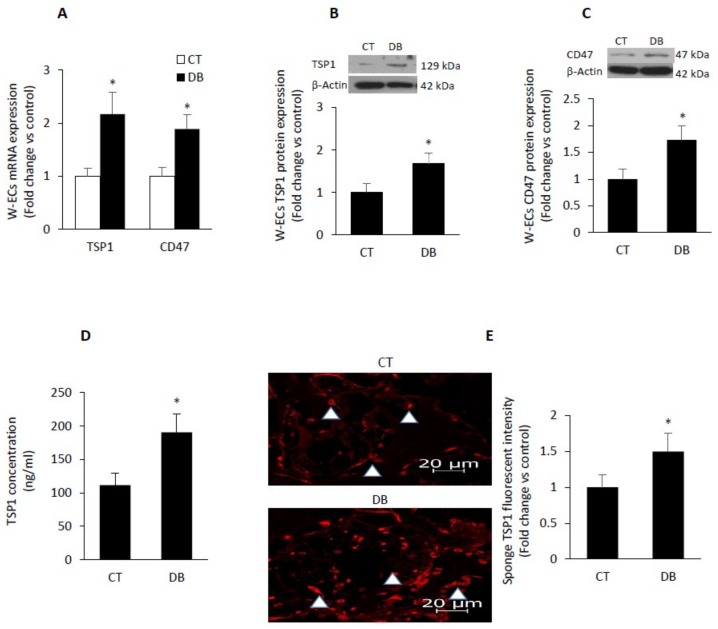

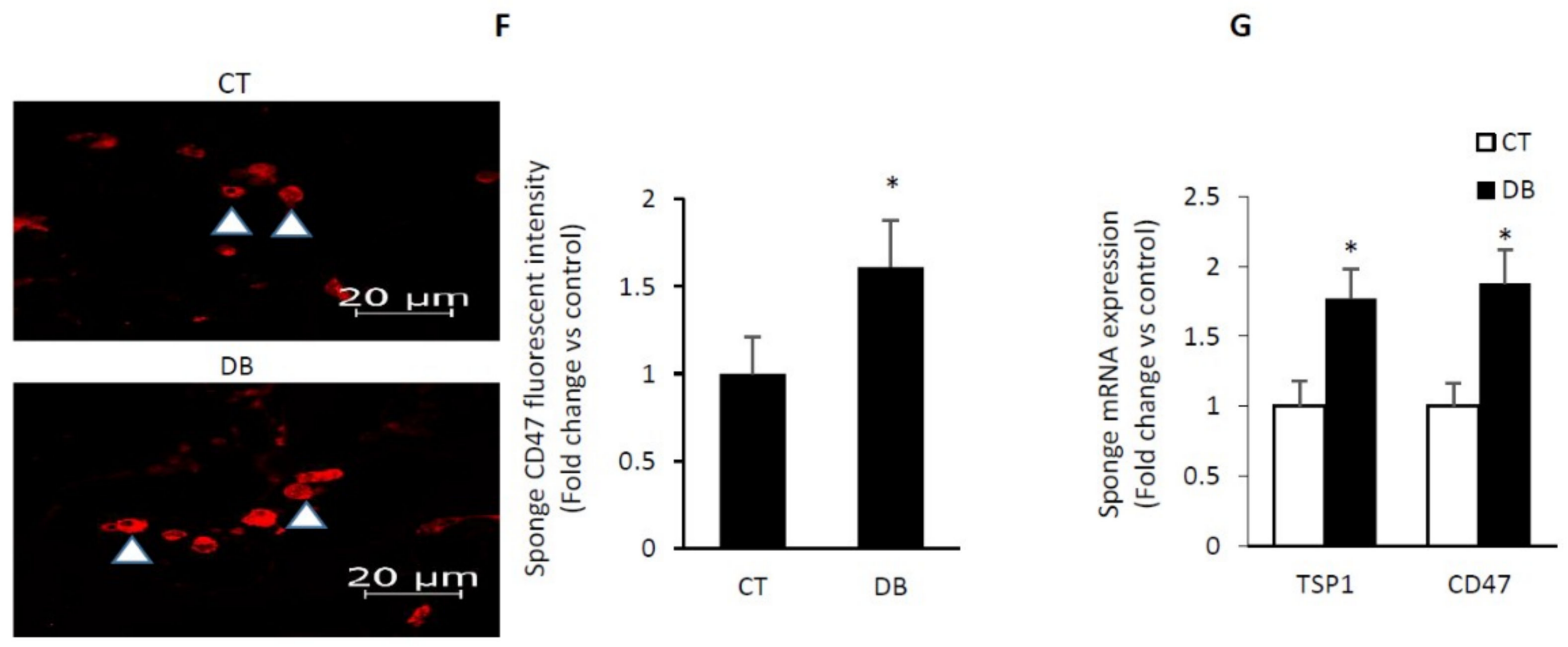
Diabetes up-regulates TSP1-CD47-dependent signaling in both W-ECs and in PVA sponge model of wound healing. (**A**–**C**) mRNA and protein levels of TSP1 and CD47 were assessed in control and diabetic W-ECs using qRT-PCR and Western blotting-based technique. (**D**) Rate of TSP1 production in cultured W-ECs was quantified using ELISA-based assay. Representative photomicrographs of TSP-1 (**E**) or CD47 (**F**) immunofluorescence staining, accompanied by a barograph figure denoting the quantitative measure of TSP-1 (**E**) or CD47 (**F**) fluorescence intensity. (**G**) mRNA expression of TSP-1 and CD47 in sponge model of wound healing was measured using qRT-PCR. Abbreviation: TSP1, thrombospondin-1 W-ECs, wound endothelial cells, CT, control; DB, diabetic. Values are means ± SEM (*n* = 6) * Significantly different from corresponding control values at *p* ≤ 0.05.

A number of studies have suggested that plasma TSP1 levels positively correlated with increased production of ROS and also with the severity of vascular disease [43,44]. However, data regarding TSP1 and its receptor CD47 during the course diabetes are somewhat limited. Accordingly, we assessed both in vitro (W-ECs) and in vivo (PVA sponge) the level of this well -known anti antiangiogenic system. Our findings confirmed that diabetic W-ECs demonstrated increased expression of TSP1 and CD47 both at the mRNA and protein levels (Figure 6A–C). Further, we also assessed the rate of production of TSP1 in cultured endothelial cells and found it to be also enhanced as a function of diabetes (Figure 6D). Interestingly, this overexpression of TSP1-CD47-dependent signaling in diabetic endothelial cells was recapitulated in the in vivo PVA sponge model of wound healing (Figure 6E–G). It is worthy of note that TSP1 interacts not only with CD47 to inhibit angiogenesis but also with other pro-angiogenic factors such as VEGF and fibroblast growth factor 2 (FGF-2), preventing them from binding to their high affinity receptors such as VEGFR2 and FGFR1 [45,46].

### 2.7. Diabetes Impairs Angiogenesis and Induces Endothelial Cell Senescence by Up-Regulating TSP1-CD47-Depndent Signaling

**Figure 7:**
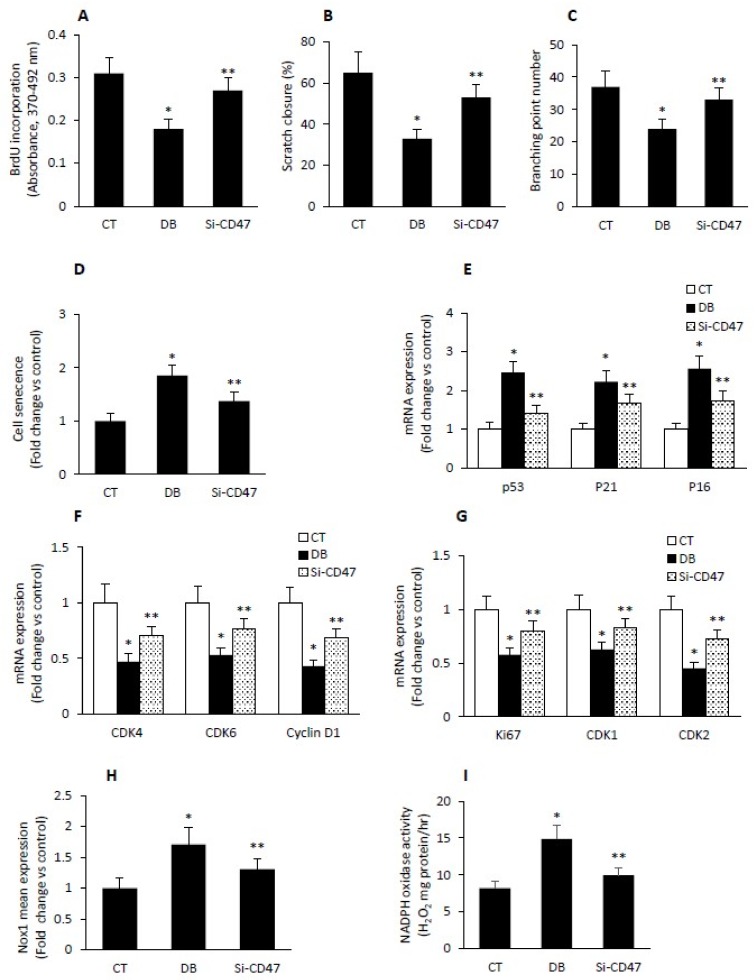
Diabetes impairs angiogenesis and induces endothelial cell senescence/oxidative stress by up-regulating TSP1-CD47-depndent signaling. Angiogenic capacity as reflected by cell proliferation (**A**), cell migration (**B**) and tube formation (**C**) was measured in cultured diabetic W-ECs harboring either CD47-siRNA or its corresponding scrambled siRNA. (**D**) Senescence as reflected by SA-β-gal activity was determined in in cultured W-ECs harboring either CD47-siRNA or its corresponding scrambled siRNA. Cell cycle regulators including CKIs (**E**); p53, p21, p16), cell cycle promoters (**F**); Ki67, CDK1, CDK2) and S-phase gene transcription (**G**); CDK4, CDK6, Cyclin D1) were quantified in cultured W-ECs harboring either CD47-siRNA or its corresponding scrambled siRNA using qRT-PCR-based assay. Oxidative stress, as reflected by Nox1 mRNA expression (**H**) or NADPH oxidase activity (**I**) was quantified in cultured W-ECs harboring either CD47-siRNA or its corresponding scrambled siRNA using qRT-PCR-based assay (e.g., Nox1 mRNA) or the 100,000× *g* cell fraction and the fluorescence probe Amplex red (NADPH oxidase activity). Abbreviation: W-ECs, wound endothelial cells; CKIs, cell cycle inhibitors, CK, cyclin-depend kinase; CT control; DB, diabetic; Si-CD47, diabetic wound endothelial cells-treated with CD47-siRNA. Values are means ± SEM (*n* = 6) * Significantly different from corresponding control values at *p* ≤ 0.05. ** Significantly different from corresponding diabetic control receiving scrambled siRNA at *p* ≤ 0.05.

Next, we examined during the course of diabetes whether a cause-and-effect relationship exists between the overexpression of TSP1/CD47-dependent signaling and the reduction in endothelial angiogenic activity. Further, the phenomenon of senescence in endothelial cells was also considered in this study. A loss-of-function strategy involving CD47 siRNA was employed in diabetic W-ECs. Transfection efficiency was confirmed with real-time PCR and western blotting. Our data revealed that the angiogenic capacity expressed in terms of endothelial cell proliferation, migration and tube formation was higher in diabetic W-ECs harboring the siRNA against CD47 than corresponding cells receiving only scrambled siRNA (Figure 7A–C). These findings harmonize with previous observations demonstrating that CD47 is a critical cell surface receptor for the antiangiogenic activity of TSP1 even at physiological concentration [28].

Additional experimentations were conducted to illustrate the involvement of TSP1-CD47-depndent signaling in the induction of endothelial senescence as a function of diabetes. In this connection, we have shown that SA-β-gal activity, a hallmark of cellular senescence together with CKIs p53, p21 and p16 were reduced in CD47- siRNA-treated diabetic W-ECs relative to diabetic W-ECs receiving only the scrambled siRNA (Figure 7D,E). In contrast, cell cycle promoters (e.g., CDK4, CDK6 and Cyclin D1) and the S-phase gene transcription (e.g., CDK1, CDK2 and Ki67) were up-regulated in CD-47 deficient diabetic W-ECs (Figure 7F,G). Finally, the heightened state of oxidative stress as indicated by increased ROS generation and Nox1 expression in diabetic W-EC was also mitigated by down-regulating the TSP1 receptor CD47 (Figure 7H,I). Overall, our data point to the possibility that the enhancement in TSP1-CD47-dependent pathway in diabetic W-ECs contributed at least in part for increased ROS generation, impaired angiogenic activity, and the induction of cell cycle arrest in connection with cellular senescence.

## 3. Discussion

A decrease angiogenesis capacity has been implicated in diabetic non-healing chronic wounds and cardiovascular disorders and suggested as a therapeutic target [30,31,32,33]. Nevertheless, the underlying molecular mechanisms remain incompletely defined. The current study contributes to filling this gap by disclosing the role of TSP1-CD47 signaling in cellular senescence and impaired angiogenesis.

Herein we report that diabetes impaired the in vivo sponge angiogenic capacity by decreasing fibrovascular invasion, capillary density, and blood vessel maturation. In contrast, a heightened state of oxidative stress and elevated expression of TSP1 and CD47 both at the mRNA and protein levels were evident in this diabetic sponge model of wound healing. These data were recapitulated using in vitro culturing system involving diabetic W-ECs. Interestingly, the diabetic W-ECs also exhibited a characteristic feature of cellular senescence. Indeed, increased SA-β-gal activity and cell cycle arrest were confirmed in these cells. The functional consequence of this cascade of events was illustrated by a marked reduction in diabetic endothelial cell proliferation, migration and tube formation. A genetic-based strategy in diabetic W-ECs using CD47 siRNA significantly ameliorated most of the aforementioned abnormalities.

Angiogenesis, a process that occurs during the course of tissue healing and cardiac hypertrophy, appears to be altered as a function of diabetes [47]. This phenomenon is regulated by a delicate balance between endogenous pro-angiogenic and anti-angiogenic molecules. Our current and previous data [48] in W-ECs and PVA sponges revealed that in conditions such as diabetes, this angiogenic balance is altered in a manner consistent with an over-production of the angiostatic factor, TSP1 and a down-regulation of the expression of the pro-angiogenic molecule, VEGF. This duality of effect of diabetes as a suppressor of VEGF and an inducer of TSP1 is reminiscent of those reported previously in aged animals [49] and in decorin-overexpressed cultured endothelial cells [50].

Endothelial cell senescence encompassing cell cycle arrest and secretion of pro-inflammatory mediators may contribute to diabetes-related decrease of both cellular replicative capacity and angiogenic activity. In the present work, we showed that diabetic W-ECs at passage 6 exhibited a phenotypic feature of cellular senescence as exemplified by enhanced SA-β-gal activity, cell cycle arrest and increased expression of SASP including TNF-α, IGFBP5, IL6 and IL8. These findings are not dissimilar from those reported previously in wound diabetic fibroblasts [51] and certain other cell types [52,53,54].

ROS at normal concentrations mediate cellular functions and signal transduction [55]. However, a mild chronic increase in ROS may trigger tissue accumulation of oxidized macromolecules and this event of oxidative damage has been advanced as one of the major determinant of cellular senescence [56], an inexorable cell fate in mammals and other organisms [57,58]. Our current data are consistent with the free radical theory of aging in two ways. We discovered that diabetic W-ECs exhibited a heightened state of oxidative stress as exemplified by the following: enhancement in NADPH oxidase activity, augmented expression of the NADPH oxidase isoform Nox1, increased DHE staining, a marker of ROS generation and finally reduced glutathione level, an indicator of endogenous anti-oxidant capacity (Un-published observation). Second, the senescent phenotypic feature of diabetic W-ECs was prevented by the anti-oxidant N-acetyl cysteine and the well-known activator of Nrf2, sulforaphane (unpublished data). To this end, our findings are not without precedent since pervious authors have reported that premature senescence of endothelial cell upon chronic exposure to TNF-α was associated with increased ROS and this phenomenon was mitigated with N-acetyl cysteine [16].

A panoply of evidence consider TSP1 as a potent inhibitor of angiogenesis and this antiangiogenic effect appears to be mediated by its receptors CD36 and CD47 [28,59,60,61]. In this context, TSP1 abundance inversely correlated with proliferation in some cell types [62]. Further, the lack of CD47 expression in endothelial cells enabled these cells to spontaneously gain characteristics of embryonic stem cells [30]. More recent reports revealed that TSP1 promotes ROS generation via the up-regulation of Nox1 [63]. The current data confirmed that the process of angiogenesis was inhibited as a function of diabetes both in in vitro and in vivo sponge model of wound healing. Similarly, we also found that diabetic W-ECs displayed phenotypic features that are consistent with heightened state of oxidative stress, increased production of pro-inflammatory cytokines and enhanced activity of SA-β-gal, a key marker of cellular senescence. To this end, we proposed that impaired angiogenesis and senescence of W-ECs during the course of diabetes may stem, at least in part, from enhanced TSP1-CD47-depdent signaling. Indeed, our data showing increased expression of TSP1 and CD47 both at the mRNA and protein levels give credence to this suggestion. More importantly, a genetic -based strategy involving the down-regulation of CD47 using siRNA increased diabetic endothelial angiogenic capacity, decreased Nox-1-mediated ROS generation, suppressed activation of cell cycle inhibitors, upregulated cell cycle promoters and finally enhanced cell cycle progression and reduced SA-β-gal activity. Consistent with these findings are the previous reports confirming that human endothelial cell lacking CD47 showed marked improvement in cell cycle progression, delayed replicative senescence, improved angiogenic potential and reduced expression of Nox1 [31,63]. Interestingly and worthy of note is the recent data showing that the TSP-Hep1 peptide located in the N-terminal domain of TSP-1 exhibited proaigiogenic effect on endothelial cells [64]. In addition, this peptide also potentiated fibroblast growth factor-2-indued neovascularization [64]. Similarly, TSP-1-derived peptide RFYVVMWK improved the adhesive phenotype of CD34 positive cells from atherosclerotic patients with type 2 diabetes [65]. To this end, the dual role of TSP-1in angiogenesis might depends on its metabolism within a cell microenvironment yielding smaller peptide molecules that affect endothelial cell functions in an opposite manner. Accordingly, the complex TSP-1 interactome and its context-dependent role suggest that the use of a predicative system biology model would greatly aid not only in the understanding of its diverse functions but also in laying the basis for the rational design of TSP-1-based therapy

Overall, the current data linking endothelial/PVA sponge TSP1-CD47-depndent signaling to cellular senescence, impaired angiogenesis and to the heightened state of oxidative stress during the course of diabetes are of value from both a mechanistic and a pharmacological standpoint. Moreover, they illuminate Nox1 and TSP1-CD47 axis as attractive therapeutic targets for the treatment of non-healing diabetic wounds and various cardiovascular disorders associated with endothelial dysfunction and reduced angiogenic activity.

## 4. Materials and Methods

### 4.1. Animals and an In Vivo Sponge Model of Angiogenesis

Goto Kakizaki (GK; age 12–15 months) female rats weighing 275-325 g, a model for non-obese type 2 diabete and their Wistar control counterparts with a similar body weight were used for this experiment. The animals were housed under diurnal lighting conditions and fed on a laboratory diet with water. Animal experiments were conducted according to the guidelines for Principles of Laboratory Animal Care and the Guide for the Care and Use of Laboratory Animals (NIH publication No 85-23, revised 1996). The current investigation was approved by the local institutional review board of Kuwait University, Faculty of Medicine. 

Polyvinyl alcohol (PVA) sponge implanted subcutaneously is a common in vivo experimental approach to assess cellular, and molecular processes associated with angiogenesis; it was used in the current study to evaluate the in vivo angiogenic capacity in GK rats. Unlike Matrigel [66], the PVA sponges maintain their shape and size for up to 4 weeks. Age and sex matched Wistar rats were used as corresponding controls. We have extensive experience using GK rats in assessing a number of cardiovascular and wound healing related events both in vivo and in vitro [6,51,67,68]. The general procedure for the PVA sponge insertion has been described previously by us and others [69,70]. Briefly, sterile PVA sponges (Pharmacia & Upjohn, Peapack, NJ, USA) were cut into pieces and hydrated overnight at 4 °C in sterile phosphate-buffered saline (PBS). Excess PBS was removed by blotting. Animals were anesthetized by an intraperitoneal injection of Ketamin/zylazine and the dorsal surface was shaved, cleaned with alcohol and incised at the midline. Four subcutaneous pockets were made on either side of the incision (2–3 cm away from the incision) with blunt-ended forceps. One sponge was inserted in each pocket before closing the incision with 6-0 sutures (Fine Science Tools, Foster City, CA, USA). Ten days later, the rats were euthanized and the PVA sponges were removed, washed once with PBS, and then either immersion fixed overnight in 10% buffered formalin for histochemical evaluation or frozen in OCT compound and/or liquid nitrogen for immune-fluorescence microscopy or various biochemical and molecular assays.

### 4.2. GTT and MOMA-IR Assessment

Oral glucose tolerance test (GTT) was determined by administering glucose intraperitoneally to Wistar control and GK rats at a dose of 2 g/kg and then blood glucose was measured at 0, 15, 30, 60, and 120 min. Similarly, homeostasis model assessment of insulin resistance (HOMA-IR) was calculated from the equation (insulin (mU/L) × glucose (mmol/L) × 22.5) using fasting blood glucose and fasting insulin levels [71].

### 4.3. Assessment of, Fibroblast Invasion, Cell Proliferation and Microvascular Density in Sponge Implants

Hematoxylin-eosin stained sponge sections were evaluated microscopically for fibrovascular invasion. Similarly, cell proliferation [54] and microvascular density was calculated in PCNA and CD31-stained sponge sections using an immuno-fluorescence-based technique. 

### 4.4. W-ECs as an In Vitro Model of Angiogenesis

An overnight PBS hydrated sponge containing endothelial cell growth supplement was inserted subcutaneously in an anesthetized rat. Eight to ten days later, the sponges were removed aseptically and used for the isolation of W-ECs as described previously [72]. The isolated cells were cultured under standardized condition (37 °C, 5% CO_2_) in 25-cm^2^ flasks precoated with 1% gelatin (type B from bovine skin, Sigma, Kawasaki, Japan) in 20% FCS-DMEM containing 20 mM HEPES, sodium pyruvate, freshly added heparin and endothelial cell growth supplement. Confluent cells were passed routinely at a split ratio 1:3 and then cultured as described above.

### 4.5. Assessment of Key W-EC Functions

#### 4.5.1. Cell Proliferation

To assess endothelial cell proliferation and cell division index, WST-1 (Dojindo, Rockville, MD, USA), BrdU incorporation (Roche Diagnostics, Floham Park, NJ USA; and Carboxyfluorescein succinimidyl ester (CFSE) dilution assay (ThermoFisher Scientic, Waltham, MA, USA) were used according to the manufacturer’s instructions. For WST-1 and BrdU assays, W-ECs of 100 µL suspension were seeded into a 96-well plate at a density of 5 × 10^3^ or 1 × 10^4^ per well and then were allowed to adhere overnight in 10% FCS-DMEM medium containing growth supplements. After being subjected to various manipulations, absorbance was measured either at 450 nm, for WST-1 or at 370–492 nm for BrdU. For CFSE assay, W-ECs were stained with CFSE at a final concentration of 2 µM in PBS/0.1% BSA for 10 min at 37 °C, washed with ice-cold DMEM with 0.1% FBS and then measured using flow cytometry; the data were expressed in terms of division index.

#### 4.5.2. Wound Healing Assay

For the in vitro wound (migration) experiments, cultured W-ECs were grown in six-well plates until they reached confluence. The medium was removed; cells were washed 3× with PBS and the culturing process was continued in serum-free DMEM containing 0.5% BSA for an additional 24 h. Thereafter, the monolayer was artificially wounded by using a pipette tip to scratch across the plate; the cells were washed with PBS to remove the detached cells and then cultured in medium containing mitomyocin C (10 µg/mL) to prevent cell proliferation. The rate of wound healing was quantified according to our previously published procedure [73].

#### 4.5.3. Tube Formation Assay

Tube formation assay was conducted by initially coating a 96-well plate with 50 µL/well growth factor reduced Matrigel (BD Biosciences), followed by incubation for 30 min at 37 °C to allow gel polymerization. ECs (1.5 × 10^4^) were seeded into each Matrigel-coated well in the presence of appropriate treatment. Image of forming tubes were captured after 12 h of incubation using a Spot camera equipped inverted microscope. The branching area of the tube network in the entire field of each well was calculated with Wimasis Image Analysis (Ibidi). 

### 4.6. Assessment of mRNA Expression, Protein Levels and ROS Contents in Sponge Implants and W-ECs

#### 4.6.1. Real-Time PCR for mRNA Quantitation

Total RNA from cells or frozen sponge implants was extracted using the Trizol reagent (Invitrogen), and the RNA integrity was verified by agarose gel electrophoresis. Approximately 1 µg of RNA was reversed transcribed (Superscript II Reverse Transcriptase Kit, Invitrogen) and amplified using the TaqMan Assay on Demand (Applied Bio systems, Foster City, CA, USA) in a 25 µL reaction volume containing two unlabeled primers, a 6-carboxyfluorescien-labeled TaqMan MGB probe and the master mix. The amplified sequences were assessed using the ABI 7500 Prism Sequence Detection system machine. The results were expressed as mRNA levels normalized to 18S or GAPDH in each sample.

#### 4.6.2. Western Blot

Cells or sponge implants were sonicated on ice in RIPA buffer containing 1% NP-40, 0.5% deoxycholate and a protease/phosphatase inhibitor cocktail (Roche Diagnostics) and the resulting homogenates were centrifuged at 15,000× *g* for 15 min at 4 °C. Proteins derived from total lysates were loaded onto an SDS-polyacrylamide gel and transferred to a PVDF membrane (Bio-Rad, Hercules, CA, USA). The membranes were blocked and then incubated with the primary antibody diluted in 5% non-fat dry milk in TBST buffer (10 mM Tris-HCl, pH 7.5, 150 mM NaCl, 0.05% Tween 20) overnight at 4 °C. After washing, the blots were incubated with the secondary antibody conjugated to HRP in TBST for 1 h at room temperature. The proteins were visualized with a Super Signal West Pico-Chemiluminescent Substrate (Pierce, Rockford, IL, USA) according to the manufacturer’s protocol. 

#### 4.6.3. Immunofluorescence Imaging

Paraffin or OCT sponge sections and coverslip-paraformaldehyde-fixed ECs were permeabilized with 0.1% Triton X-100 in PBS for 15 min, washed 3 times with PBS, and incubated for 1 h at room temperature in blocking buffer containing PBS and 2% BSA. Coverslips or sponge sections were incubated with a primary antibody overnight at 4 °C, followed by washing 3 times with PBS and then incubating for 1 h with the appropriate fluorescence-labeled secondary antibody. Samples were mounted in antifade medium with DAPI (Vector Laboratories). We used dihydroethidium (DHE) to examine ROS in both OCT frozen sponge sections and in ECs grown on coverslips. An LSM 510 L confocal laser scanning microscope (Carl Zeiss, Oberkochen, Germany) with Axiovert 100 M was used to analyze ROS or immune-stained samples and to capture representative Images. Confocal images were converted to 8-bit gray-scale. Images obtained were exported by Axio-Vision software (Carl Zeiss) in TIF format.

#### 4.6.4. ELISA Quantification of TSP1

Levels of TSP1, IL-6 and IL-8 in conditional medium were assessed using a commercially available kit specific for rat TSP1, (ABclonal, Manhattan Beach, CA, USA); IL-6, (R&D, Minneapolis, MN, USA); IL-8, MyBioSource, San Diego, CA, USA) according to the manufacturer’s instructions.

#### 4.6.5. siRNA Transfection

The expression of CD47 in diabetic W-ECs was genetically down-regulated by siRNA oligonucleotides. The sequences were designed and synthesized by Qiagen (Germantown, MD, USA). The day before transfection, cells were seeded in complete DMEM medium. The next day, cells were washed and then overlaid with Opti-MEM medium (Gibco, Carlsbad, CA, USA). Optimum silencing efficiency was obtained by adding 18 µL of 20 µM siRNA to 142 µL Opti-MEM medium, incubated at room temperature for 15 min, and then Oligofectamine mixture (Invitrogen, Carlsbad, CA, USA) was added according to the manufacturer’s instruction (8 µL Oligofectamine + 32 µL of Opti-MEM medium, incubated for 5 min at room temperature). Complexes were added to the cells and after 4 h of incubation in the CO_2_ incubator, DMEM containing FCS was added to the well. Following overnight incubation, the cells were washed with PBS, incubated in DMEM medium and then used in various functional and biochemical assays. Efficiency of the knockdown of CD47 was verified by real-time PCR or Western blotting.

### 4.7. Senescence-Associated β-Galactosidase (SA-β-gal) Expression Assay

SA-β-gal activity, a marker of cellular senescence, was measured in W-ECs using a light microscope staining- (Cell Signaling Technology, Danvers, MA, USA) or florescence-based assay (Cell Biolab, San Diego, CA, USA) according to manufacturer’s protocol. 

### 4.8. ROS Generation and NAD(P)H Oxidase Activity

ROS generation was determined in both PVA sponge model of wound healing and in W-ECs cultured on coverslip using the florescence probe dichlrofluorescein diacetate (DCF-DA) in connection with confocal microscopy for quantification. On the other hand, the rate of NADPH-dependent superoxide/H_2_O_2_ generation in 100,000× *g* membrane fractions of control and diabetic endothelial cells was assessed using lucigenin chemiluminescence or the Amplex red/horseradish peroxidase fluorescence assay.

### 4.9. Apoptosis Assay

Apoptosis assay was performed using the cytoplasmic histone-associated DNA fragments detection kit (Roche Applied Science) and according to the manufacture’s protocol. Briefly, W-ECs were plated in 96-well plates at a density of 1 × 10^4^/well for 48 h. Next, cells were lysed and centrifuged at 15,000× *g* for 10 min and the collected supernatants were subjected to ELISA apoptosis detection plate. The absorbance was measured at 405 nm.

### 4.10. Statistical Analysis

Data are expressed as the means ± SEM. Comparisons were made by two-tailed paired Student’s *t*-test or by one-way analysis of variance followed by Bonferroni post hoc test. A level of *p* ≤ 0.05 was considered to be significant.

## References

[1] Palmer A.K., Tchkonia T., LeBrasseur N.K., Chini E.N., Xu M., Kirkland J.L. (2015). Cellular Senescence in Type 2 Diabetes: A Therapeutic Opportunity. Diabetes.

[2] Beckman J.A., Creager M.A., Libby P. (2002). Diabetes and atherosclerosis: Epidemiology, pathophysiology, and management. Jama.

[3] Brem H., Tomic-Canic M. (2007). Cellular and molecular basis of wound healing in diabetes. J. Clin. Investig..

[4] Kannel W.B., McGee D.L. (1979). Diabetes and glucose tolerance as risk factors for cardiovascular disease: The Framingham study. Diabetes Care.

[5] Jeffcoate W.J., Harding K.G. (2003). Diabetic foot ulcers. Lancet.

[6] Bitar M.S., Ayed A.K., Abdel-Halim S.M., Isenovic E.R., Al-Mulla F. (2010). Inflammation and apoptosis in aortic tissues of aged type II diabetes: Amelioration with alpha-lipoic acid through phosphatidylinositol 3-kinase/Akt-dependent mechanism. Life Sci..

[7] Bitar M.S., Wahid S., Mustafa S., Al-Saleh E., Dhaunsi G.S., Al-Mulla F. (2005). Nitric oxide dynamics and endothelial dysfunction in type II model of genetic diabetes. Eur. J. Pharmacol..

[8] Tahergorabi Z., Khazaei M. (2012). Imbalance of angiogenesis in diabetic complications: The mechanisms. Int. J. Prev. Med..

[9] Falanga V. (2005). Wound healing and its impairment in the diabetic foot. Lancet.

[10] Galiano R.D., Tepper O.M., Pelo C.R., Bhatt K.A., Callaghan M., Bastidas N., Bunting S., Steinmetz H.G., Gurtner G.C. (2004). Topical vascular endothelial growth factor accelerates diabetic wound healing through increased angiogenesis and by mobilizing and recruiting bone marrow-derived cells. Am. J. Pathol..

[11] Waltenberger J. (2009). VEGF resistance as a molecular basis to explain the angiogenesis paradox in diabetes mellitus. Biochem. Soc. Trans..

[12] Minamino T., Miyauchi H., Yoshida T., Ishida Y., Yoshida H., Komuro I. (2002). Endothelial cell senescence in human atherosclerosis: Role of telomere in endothelial dysfunction. Circulation.

[13] Counter C.M., Avilion A.A., LeFeuvre C.E., Stewart N.G., Greider C.W., Harley C.B., Bacchetti S. (1992). Telomere shortening associated with chromosome instability is arrested in immortal cells which express telomerase activity. EMBO J..

[14] Valenzuela M.T., Nunez M.I., Villalobos M., Siles E., McMillan T.J., Pedraza V., Ruiz de Almodovar J.M. (1997). A comparison of p53 and p16 expression in human tumor cells treated with hyperthermia or ionizing radiation. Int. J. Cancer.

[15] Matsui-Hirai H., Hayashi T., Yamamoto S., Ina K., Maeda M., Kotani H., Iguchi A., Ignarro L.J., Hattori Y. (2011). Dose-dependent modulatory effects of insulin on glucose-induced endothelial senescence in vitro and in vivo: A relationship between telomeres and nitric oxide. J. Pharmacol. Exp. Ther..

[16] Khan S.Y., Awad E.M., Oszwald A., Mayr M., Yin X., Waltenberger B., Stuppner H., Lipovac M., Uhrin P., Breuss J.M. (2017). Premature senescence of endothelial cells upon chronic exposure to TNFalpha can be prevented by N-acetyl cysteine and plumericin. Sci. Rep..

[17] Corre I., Paris F., Huot J. (2017). The p38 pathway, a major pleiotropic cascade that transduces stress and metastatic signals in endothelial cells. Oncotarget.

[18] Munoz-Espin D., Serrano M. (2014). Cellular senescence: From physiology to pathology. Nat. Rev. Mol. Cell Biol..

[19] Childs B.G., Durik M., Baker D.J., van Deursen J.M. (2015). Cellular senescence in aging and age-related disease: From mechanisms to therapy. Nat. Med..

[20] Haendeler J., Hoffmann J., Diehl J.F., Vasa M., Spyridopoulos I., Zeiher A.M., Dimmeler S. (2004). Antioxidants inhibit nuclear export of telomerase reverse transcriptase and delay replicative senescence of endothelial cells. Circ. Res..

[21] Matsushita H., Chang E., Glassford A.J., Cooke J.P., Chiu C.P., Tsao P.S. (2001). eNOS activity is reduced in senescent human endothelial cells: Preservation by hTERT immortalization. Circ. Res..

[22] Xin M.G., Zhang J., Block E.R., Patel J.M. (2003). Senescence-enhanced oxidative stress is associated with deficiency of mitochondrial cytochrome c oxidase in vascular endothelial cells. Mech. Ageing Dev..

[23] Yoon H.J., Cho S.W., Ahn B.W., Yang S.Y. (2010). Alterations in the activity and expression of endothelial NO synthase in aged human endothelial cells. Mech. Ageing Dev..

[24] Lakatta E.G., Levy D. (2003). Arterial and cardiac aging: Major shareholders in cardiovascular disease enterprises: Part I: Aging arteries: A “set up” for vascular disease. Circulation.

[25] Rivard A., Fabre J.E., Silver M., Chen D., Murohara T., Kearney M., Magner M., Asahara T., Isner J.M. (1999). Age-dependent impairment of angiogenesis. Circulation.

[26] Zhao C., Isenberg J.S., Popel A.S. (2018). Human expression patterns: Qualitative and quantitative analysis of thrombospondin-1 under physiological and pathological conditions. J. Cell. Mol. Med..

[27] Zaslavsky A., Baek K.H., Lynch R.C., Short S., Grillo J., Folkman J., Italiano J.E., Ryeom S. (2010). Platelet-derived thrombospondin-1 is a critical negative regulator and potential biomarker of angiogenesis. Blood.

[28] Isenberg J.S., Ridnour L.A., Dimitry J., Frazier W.A., Wink D.A., Roberts D.D. (2006). CD47 is necessary for inhibition of nitric oxide-stimulated vascular cell responses by thrombospondin-1. J. Biol. Chem..

[29] Sick E., Jeanne A., Schneider C., Dedieu S., Takeda K., Martiny L. (2012). CD47 update: A multifaceted actor in the tumour microenvironment of potential therapeutic interest. Br. J. Pharmacol..

[30] Kaur S., Soto-Pantoja D.R., Stein E.V., Liu C., Elkahloun A.G., Pendrak M.L., Nicolae A., Singh S.P., Nie Z., Levens D. (2013). Thrombospondin-1 signaling through CD47 inhibits self-renewal by regulating c-Myc and other stem cell transcription factors. Sci. Rep..

[31] Gao Q., Chen K., Gao L., Zheng Y., Yang Y.G. (2016). Thrombospondin-1 signaling through CD47 inhibits cell cycle progression and induces senescence in endothelial cells. Cell Death Dis..

[32] Drinkwater S.L., Smith A., Burnand K.G. (2002). What can wound fluids tell us about the venous ulcer microenvironment?. Int. J. Low. Extrem. Wounds.

[33] Yagihashi S., Tonosaki A., Yamada K., Kakizaki M., Goto Y. (1982). Peripheral neuropathy in selectively-inbred spontaneously diabetic rats: Electrophysiological, morphometrical and freeze-replica studies. Tohoku J. Exp. Med..

[34] Sato N., Komatsu K., Kurumatani H. (2003). Late onset of diabetic nephropathy in spontaneously diabetic GK rats. Am. J. Nephrol..

[35] Sone H., Kawakami Y., Okuda Y., Sekine Y., Honmura S., Matsuo K., Segawa T., Suzuki H., Yamashita K. (1997). Ocular vascular endothelial growth factor levels in diabetic rats are elevated before observable retinal proliferative changes. Diabetologia.

[36] Rodier F., Campisi J. (2011). Four faces of cellular senescence. J. Cell Biol..

[37] Coppe J.P., Patil C.K., Rodier F., Sun Y., Munoz D.P., Goldstein J., Nelson P.S., Desprez P.Y., Campisi J. (2008). Senescence-associated secretory phenotypes reveal cell-nonautonomous functions of oncogenic RAS and the p53 tumor suppressor. PLoS Biol..

[38] Kuilman T., Michaloglou C., Mooi W.J., Peeper D.S. (2010). The essence of senescence. Genes Dev..

[39] Collado M., Blasco M.A., Serrano M. (2007). Cellular senescence in cancer and aging. Cell.

[40] Leong W.F., Chau J.F., Li B. (2009). p53 Deficiency leads to compensatory up-regulation of p16INK4a. Mol. Cancer Res..

[41] Kovac S., Angelova P.R., Holmstrom K.M., Zhang Y., Dinkova-Kostova A.T., Abramov A.Y. (2015). Nrf2 regulates ROS production by mitochondria and NADPH oxidase. Biochim. Biophys. Acta.

[42] Wingler K., Altenhoefer S.A., Kleikers P.W., Radermacher K.A., Kleinschnitz C., Schmidt H.H. (2012). VAS2870 is a pan-NADPH oxidase inhibitor. Cell. Mol. Life Sci..

[43] Novelli E.M., Little-Ihrig L., Jorgensen D.R., Shaaban C.E., Isenberg J.S., Butters M.A., Rosano C. (2015). Elevated Plasma Thrombospondin-1 (TSP1) Levels Are Correlated with Oxidative Stress and Worse Cognitive Function in Sickle Cell Disease. Blood.

[44] Smadja D.M., d’Audigier C., Bieche I., Evrard S., Mauge L., Dias J.V., Labreuche J., Laurendeau I., Marsac B., Dizier B. (2011). Thrombospondin-1 is a plasmatic marker of peripheral arterial disease that modulates endothelial progenitor cell angiogenic properties. Arterioscler. Thromb. Vasc. Biol..

[45] Gupta K., Gupta P., Wild R., Ramakrishnan S., Hebbel R.P. (1999). Binding and displacement of vascular endothelial growth factor (VEGF) by thrombospondin: Effect on human microvascular endothelial cell proliferation and angiogenesis. Angiogenesis.

[46] Pagano K., Torella R., Foglieni C., Bugatti A., Tomaselli S., Zetta L., Presta M., Rusnati M., Taraboletti G., Colombo G. (2012). Direct and allosteric inhibition of the FGF2/HSPGs/FGFR1 ternary complex formation by an antiangiogenic, thrombospondin-1-mimic small molecule. PLoS ONE.

[47] Ergul A., Abdelsaid M., Fouda A.Y., Fagan S.C. (2014). Cerebral neovascularization in diabetes: Implications for stroke recovery and beyond. J. Cereb. Blood Flow Metab..

[48] Bitar M.S., Al-Mulla F. (2015). Upregulation of CREM/ICER suppresses wound endothelial CRE-HIF-1α-VEGF-dependent signaling and impairs angiogenesis in type 2 diabetes. Dis. Models Mechan..

[49] Sadoun E., Reed M.J. (2003). Impaired angiogenesis in aging is associated with alterations in vessel density, matrix composition, inflammatory response, and growth factor expression. J. Histochem. Cytochem..

[50] Neill T., Painter H., Buraschi S., Owens R.T., Lisanti M.P., Schaefer L., Iozzo R.V. (2012). Decorin antagonizes the angiogenic network: Concurrent inhibition of Met, hypoxia inducible factor 1alpha, vascular endothelial growth factor A, and induction of thrombospondin-1 and TIMP3. J. Biol. Chem..

[51] Bitar M.S., Abdel-Halim S.M., Al-Mulla F. (2013). Caveolin-1/PTRF upregulation constitutes a mechanism for mediating p53-induced cellular senescence: Implications for evidence-based therapy of delayed wound healing in diabetes. Am. J. Physiol. Endocrinol. Metab..

[52] Hampel B., Fortschegger K., Ressler S., Chang M.W., Unterluggauer H., Breitwieser A., Sommergruber W., Fitzky B., Lepperdinger G., Jansen-Durr P. (2006). Increased expression of extracellular proteins as a hallmark of human endothelial cell in vitro senescence. Exp. Gerontol..

[53] Yanaka M., Honma T., Sato K., Shinohara N., Ito J., Tanaka Y., Tsuduki T., Ikeda I. (2011). Increased monocytic adhesion by senescence in human umbilical vein endothelial cells. Biosci. Biotechnol. Biochem..

[54] Rombouts C., Aerts A., Quintens R., Baselet B., El-Saghire H., Harms-Ringdahl M., Haghdoost S., Janssen A., Michaux A., Yentrapalli R. (2014). Transcriptomic profiling suggests a role for IGFBP5 in premature senescence of endothelial cells after chronic low dose rate irradiation. Int. J. Radiat. Biol..

[55] Zhang J., Wang X., Vikash V., Ye Q., Wu D., Liu Y., Dong W. (2016). ROS and ROS-Mediated Cellular Signaling. Oxid. Med. Cell. Longev..

[56] Kurz D.J., Decary S., Hong Y., Trivier E., Akhmedov A., Erusalimsky J.D. (2004). Chronic oxidative stress compromises telomere integrity and accelerates the onset of senescence in human endothelial cells. J. Cell Sci..

[57] Van Deursen J.M. (2014). The role of senescent cells in ageing. Nature.

[58] Vigneron A., Vousden K.H. (2010). p53, ROS and senescence in the control of aging. Aging.

[59] Jimenez B., Volpert O.V., Reiher F., Chang L., Munoz A., Karin M., Bouck N. (2001). c-Jun N-terminal kinase activation is required for the inhibition of neovascularization by thrombospondin-1. Oncogene.

[60] Isenberg J.S., Jia Y., Fukuyama J., Switzer C.H., Wink D.A., Roberts D.D. (2007). Thrombospondin-1 inhibits nitric oxide signaling via CD36 by inhibiting myristic acid uptake. J. Biol. Chem..

[61] Kanda S., Shono T., Tomasini-Johansson B., Klint P., Saito Y. (1999). Role of thrombospondin-1-derived peptide, 4N1K, in FGF-2-induced angiogenesis. Exp. Cell Res.

[62] Isenberg J.S., Ridnour L.A., Perruccio E.M., Espey M.G., Wink D.A., Roberts D.D. (2005). Thrombospondin-1 inhibits endothelial cell responses to nitric oxide in a cGMP-dependent manner. Proc. Natl. Acad. Sci. USA.

[63] Meijles D.N., Sahoo S., Al Ghouleh I., Amaral J.H., Bienes-Martinez R., Knupp H.E., Attaran S., Sembrat J.C., Nouraie S.M., Rojas M.M. (2017). The matricellular protein TSP1 promotes human and mouse endothelial cell senescence through CD47 and Nox1. Sci. Signal..

[64] Dias J.V., Benslimane-Ahmim Z., Egot M., Lokajczyk A., Grelac F., Galy-Fauroux I., Juliano L., Le-Bonniec B., Takiya C.M., Fischer A.M. (2012). A motif within the N-terminal domain of TSP-1 specifically promotes the proangiogenic activity of endothelial colony-forming cells. Biochem. Pharmacol..

[65] Cointe S., Rheaume E., Martel C., Blanc-Brude O., Dube E., Sabatier F., Dignat-George F., Tardif J.C., Bonnefoy A. (2017). Thrombospondin-1-Derived Peptide RFYVVMWK Improves the Adhesive Phenotype of CD34(+) Cells From Atherosclerotic Patients With Type 2 Diabetes. Cell Transplant..

[66] Auerbach R., Akhtar N., Lewis R.L., Shinners B.L. (2000). Angiogenesis assays: Problems and pitfalls. Cancer Metast. Rev..

[67] Bitar M.S. (2012). The GSK-3β/Fyn/Nrf2 pathway in fibroblasts and wounds of type 2 diabetes: On the road to an evidence-based therapy of non-healing wounds. Adipocyte.

[68] Bitar M.S., Al-Mulla F. (2011). A defect in Nrf2 signaling constitutes a mechanism for cellular stress hypersensitivity in a genetic rat model of type 2 diabetes. Am. J. Physiol. Endocrinol. Metab..

[69] Bitar M.S. (2000). Insulin and glucocorticoid-dependent suppression of the IGF-I system in diabetic wounds. Surgery.

[70] Bradshaw A.D., Reed M.J., Carbon J.G., Pinney E., Brekken R.A., Sage E.H. (2001). Increased fibrovascular invasion of subcutaneous polyvinyl alcohol sponges in SPARC-null mice. Wound Repair Regen..

[71] Mather K. (2009). Surrogate measures of insulin resistance: Of rats, mice, and men. Am. J. Physiol. Endocrinol. Metab..

[72] Dong Q.G., Bernasconi S., Lostaglio S., De Calmanovici R.W., Martin-Padura I., Breviario F., Garlanda C., Ramponi S., Mantovani A., Vecchi A. (1997). A general strategy for isolation of endothelial cells from murine tissues. Characterization of two endothelial cell lines from the murine lung and subcutaneous sponge implants. Arterioscler. Thromb. Vasc. Biol..

[73] Al-Mulla F., Leibovich S.J., Francis I.M., Bitar M.S. (2011). Impaired TGF-beta signaling and a defect in resolution of inflammation contribute to delayed wound healing in a female rat model of type 2 diabetes. Mol. Biosyst..

